# Secreted Amyloid Precursor Protein-Alpha Enhances LTP Through the Synthesis and Trafficking of Ca^2+^-Permeable AMPA Receptors

**DOI:** 10.3389/fnmol.2021.660208

**Published:** 2021-04-01

**Authors:** Rhys W. Livingstone, Megan K. Elder, Anurag Singh, Courteney M. Westlake, Warren P. Tate, Wickliffe C. Abraham, Joanna M. Williams

**Affiliations:** ^1^Department of Anatomy, Brain Health Research Centre, Brain Research New Zealand – Rangahau Roro Aotearoa, University of Otago, Dunedin, New Zealand; ^2^Department of Psychology, Brain Health Research Centre, Brain Research New Zealand – Rangahau Roro Aotearoa, University of Otago, Dunedin, New Zealand; ^3^Department of Biochemistry, Brain Health Research Centre, Brain Research New Zealand – Rangahau Roro Aotearoa, University of Otago, Dunedin, New Zealand

**Keywords:** secreted amyloid precursor protein alpha, FUNCAT-PLA, long-term potentiation, Alzheimer’s disease, activity related cytoskeletal (Arc) associated protein, plasticity, hippocampus, AMPA receptor

## Abstract

Regulation of AMPA receptor expression by neuronal activity and neuromodulators is critical to the expression of both long-term potentiation (LTP) and memory. In particular, Ca^2+^-permeable AMPARs (CP-AMPAR) play a unique role in these processes due to their transient, activity-regulated expression at synapses. Secreted amyloid precursor protein-alpha (sAPPα), a metabolite of the parent amyloid precursor protein (APP) has been previously shown to enhance hippocampal LTP as well as memory formation in both normal animals and in Alzheimer’s disease models. In earlier work we showed that sAPPα promotes trafficking of GluA1-containing AMPARs to the cell surface and specifically enhances synthesis of GluA1. To date it is not known whether *de novo* synthesized GluA1 form CP-AMPARs or how they contribute to sAPPα-mediated plasticity. Here, using fluorescent non-canonical amino acid tagging–proximity ligation assay (FUNCAT-PLA), we show that brief treatment of primary rat hippocampal neurons with sAPPα (1 nM, 30 min) rapidly enhanced the cell-surface expression of *de novo* GluA1 homomers and reduced levels of *de novo* GluA2, as well as extant GluA2/3-AMPARs. The *de novo* GluA1-containing AMPARs were localized to extrasynaptic sites and later internalized by sAPPα-driven expression of the activity-regulated cytoskeletal-associated protein, Arc. Interestingly, longer exposure to sAPPα increased synaptic levels of GluA1/2 AMPARs. Moreover, the sAPPα-mediated enhancement of LTP in area CA1 of acute hippocampal slices was dependent on CP-AMPARs. Together, these findings show that sAPPα engages mechanisms which specifically enhance the synthesis and cell-surface expression of GluA1 homomers, underpinning the sAPPα-driven enhancement of synaptic plasticity in the hippocampus.

## Introduction

Dynamic changes in α-amino-3-hydroxy-5-methyl-4-isoxazolepropionic acid receptor (AMPAR) expression govern neuronal synaptic efficacy, and promote synaptic plasticity. These changes are thought to underlie information coding and storage in learning and memory processes (Anggono and Huganir, 2012). Specifically, these processes are driven through the regulated synthesis and trafficking of AMPARs to and from the synapse, under strict, activity-regulated guidance (Rumpel et al., 2005). Importantly, many of these processes are negatively affected in neuropsychiatric (Maeng et al., 2008; Hammond et al., 2010; Corti et al., 2011; Kiselycznyk et al., 2013; Kabir et al., 2017; Sossin et al., 2019) and neurodegenerative disorders, including Parkinson’s disease (Chartier-Harlin et al., 2011; Cortese et al., 2016; Zhu et al., 2018), and Alzheimer’s disease **(AD;** Langstrom et al., 1989; Ding et al., 2005; Chang et al., 2006; Guntupalli et al., 2016, 2017; Garcia-Esparcia et al., 2017; Li et al., 2019). Therefore, much research has been undertaken in order to understand the role of synthesis and trafficking of AMPARs in both health and disease.

AMPARs, the primary excitatory neurotransmitter receptor in the CNS, are composed of four subunits (GluA1–GluA4), formed by a dimer of two identical homo- or heterodimers that together form the tetrameric structure. GluA1 is undoubtedly the most studied AMPAR subunit in the context of synaptic plasticity and memory. A majority of GluA1-containing AMPARs form heterodimers with GluA2 to form GluA1/2 AMPARs (Lu et al., 2009). However, GluA1 also forms GluA1 homomeric AMPARs, comprising approximately 8% of the AMPAR population in basal conditions (Wenthold et al., 1996). The absence of GluA2 renders the receptor Ca^2+^-permeable and inwardly rectifying, displaying minimal outward ion flow, extremely fast kinetics and high conductance (Geiger et al., 1995; Grosskreutz et al., 2003). Growing evidence suggests a unique contribution of Ca^2+^-permeable AMPARs (CP-AMPARs) in response to various LTP (Plant et al., 2006; Guire et al., 2008; Park et al., 2016; Yamanaka et al., 2017; Purkey et al., 2018), and LTD (Sanderson et al., 2016) stimulation paradigms. On the contrary, some groups have provided evidence to suggest that CP-AMPARs do not govern all forms of LTP (Adesnik and Nicoll, 2007; Gray et al., 2007; Guire et al., 2008; Purkey et al., 2018), and may be highly regulated by animal species and developmental age (Lu et al., 2007). Despite this, evidence for CP-AMPARs has been shown following *in vivo* learning paradigms (Clem and Barth, 2006; Conrad et al., 2008; Clem and Huganir, 2010; Hong et al., 2013; Zhang et al., 2016; Takemoto et al., 2017; Torquatto et al., 2019) and stimulation-evoked synaptic plasticity (Williams et al., 2007), and may arise in response to neuromodulators including serotonin (Jitsuki et al., 2011), nicotine (Tang et al., 2015), dopamine (Bellone and Lüscher, 2006; Gao et al., 2006), norepinephrine (Clem and Huganir, 2013), estrogen (Tada et al., 2013, 2015), tumor necrosis factor-α (Leonoudakis et al., 2008), glycine (Fortin et al., 2010; Jaafari et al., 2012), and brain-derived neurotrophic factor (Li and Keifer, 2009; Fortin et al., 2012).

Our previous work has provided evidence suggesting that the neuroprotective and neurotrophic metabolite of the transmembrane glycoprotein amyloid precursor protein (APP), secreted APP-alpha (sAPPα), may regulate similar processes. sAPPα, as well as peptide derivatives, enhances hippocampal LTP (Ishida et al., 1997; Richter et al., 2018), and rescues memory impairments (Meziane et al., 1998; Mileusnic et al., 2004; Fol et al., 2016; Xiong et al., 2017; Tan et al., 2018). We have shown that sAPPα not only promotes synaptodendritic protein synthesis (Claasen et al., 2009) and new gene transcription (Ryan et al., 2013), but brings about a protein synthesis-dependent trafficking of GluA1-AMPARs to the cell surface (Mockett et al., 2019) and enhances Arc protein expression (Livingstone et al., 2019), yet how these changes contribute to the documented sAPPα-driven enhancement of synaptic plasticity in the hippocampus is unresolved. Here, we show that sAPPα rapidly and specifically induces the synthesis of CP-AMPARs which are transported to the cell surface. Further, we show that sAPPα-mediated enhancement of LTP in area CA1 of acute hippocampal slices is dependent on CP-AMPARs. Consistent with the slot-hypothesis of AMPAR expression, the increase in cell surface GluA1 occurs alongside a reduction in cell surface GluA2/3-containing AMPARs and *de novo* GluA1-homomeric receptors are ultimately replaced by sodium channel forming-GluA1/2 receptors derived from pre-existing pools. Thus sAPPα acts as a neuromodulator by harnessing paradigmatic synaptic plasticity mechanisms, to prime the response of synapses to co-existent activity.

## Materials and Methods

### Animals

All experimental protocols were approved by the University of Otago Animals Ethics Committee and conducted in accordance with New Zealand Animal Welfare Legislation under the ethics approval ET18/15 and AUP-18-136 for cell culture work and DET19/16 for all acute slice work. All experiments conducted in primary hippocampal cultures were prepared from postnatal Sprague-Dawley rat pups (male or female, P0-P1) sourced from a breeding colony maintained at the Hercus Taieri Resource Unit by the University of Otago (Dunedin, New Zealand). The preparation of primary hippocampal cultures followed a modified protocol based on Banker and Goslin (1998) and Kaech and Banker (2006). Hippocampi were dissociated using papain (Sigma) and plated at a low density on glass-bottomed culture dishes (40,000 cells/cm^2^; Mattek), or 96-well assay plates (67,500 cells/cm^2^; Corning, #3603) for immunolabelling. Cells were cultured in Neurobasal A medium (Life Technologies #10888-022), supplemented with B27 (Life Technologies, #17504-001) and Glutamax (Life Technologies, #35050-061) at 37°C/5% CO_2_ for 21–27 days *in vitro* (DIV). Control treatments were undertaken at the same time in matched sets of culture dishes (FUNCAT-PLA, immunocytochemistry).

### Drugs and Reagents

For experiments examining the role of Ca^2+^-permeable receptors the antagonist IEM-1460 was used (100 μM, Abcam, #AB141507). sAPPα production and purification was carried out according to Turner et al. (2007).

### Preparation of siRNA

Inhibition of Arc synthesis was achieved using Acell^TM^ siRNA (1 μM, CUGCAGUACAGUGAGGGUA; Dharmacon, #A-080172-15-0020) targeted to the open-reading frame of the Arc gene, as well as the control non-targeting siRNA (1 μM, UGGUUUACAUGUGUCGACUAA; Dharmacon, #D-001910-01-20). All Accell^TM^ siRNA were prepared in a 1x siRNA reconstitution buffer consisting of (in mM): KCL 300, MgCl_2_ 1, HEPES 30 in RNAse-free water (pH 7.3–7.6), and incubated at 37°C with gentle rocking for 70 min, as according to manufacturer’s instructions. Reconstituted siRNA were aliquoted and stored at −20°C until needed. Expression of siRNA in cultured neurons was confirmed by detection of the red non-targeting (NT) control siRNA (1 μM, UGGUUUACAUGUGUCGACUAA; DY-547, Dharmacon, #D-001960-01-05).

### Primary Antibodies

For the detection of proteins *in situ* primary antibodies were used targeting Arc (rabbit polyclonal, 1:1000, synaptic systems, #156003), Biotin (mouse monoclonal, 1:1000, Sigma, #B7653), MAP2 (guinea-pig polyclonal, 1:1000, synaptic systems, #188004; monoclonal, 1:1000, Abcam, #AB11267), Synapsin-1 (mouse monoclonal, 1:1000, synaptic systems, #106011), GluA1 (C-terminal; rabbit polyclonal, 1:500, Abcam, #AB31232), GluA1 (N-terminal; mouse monoclonal, 1:500, #MAB2263), GluA2 (C-terminal; rabbit polyclonal, 1:500, Abcam, #AB1768-I), GluA2 (N-terminal; mouse polyclonal, 1:500, Abcam, #133477), and GluA3 (N-terminal; mouse monoclonal, 1:250, Thermofisher, #32-0400).

### Secondary Antibodies and Fluorescent Reagents

Detection of primary antibodies was achieved via addition of the following secondary antibodies: Goat anti-Guinea Pig Alexa Fluor 488 (1:1000, Thermofisher, #A11073), Goat anti-rabbit Alexa Fluor 555 (1:1000, Invitrogen, #A21429), Goat anti-mouse Alexa Fluor 488 (1:500; Thermofisher, #A11001), Donkey anti-mouse PLA^*minus*^ probe (1:10, Sigma-Aldrich, #DUO92004), Donkey anti-rabbit PLA^*plus*^ probe (1:10, Sigma-Aldrich, #DUO92002), DAPI (1:1000, Thermofisher, #D1306), and Duolink detection reagent Texas Red (1:5, Sigma-Aldrich, #DUO92008).

### Immunocytochemistry

For experiments examining the effect of siRNA treatment on protein expression, DIV21-27 primary hippocampal neurons were treated with sAPPα (1 nM; 2 h), or culture media only. Following pre-treatment with siRNA (1 μM, 60 min), primary hippocampal cultures were co-treated with either Arc or NT siRNA (1 μM, 2 h), in the presence or absence of sAPPα (1 nM). Following incubation, cells were fixed in 4% paraformaldehyde (pH 7.4; 20 min) in PBS supplemented with 1 mM MgCl_2_ and 0.1 mM CaCl_2_ (PBS-MC) containing sucrose (PBS-MCS; 155.42 mM), and permeabilized with 0.5% Triton X-100 in PBS (pH 7.4; 15 min; for the detection of cell surface GluA1 using an N-terminal antibody this step was omitted). Cells were then blocked in 4% normal goat serum in PBS (pH 7.4) for 60 min at room temperature (RT). Cells were incubated with primary antibodies of interest (2 h, RT), followed by 3 × 5 min washes (PBS, pH 7.4) and incubation in appropriate secondary antibody (30 min, RT), followed by 3 × 5 min washes (PBS, pH 7.4).

### FUNCAT-PLA

FUNCAT-PLA labeling of newly synthesized proteins was conducted according to a previously published protocol (Dieterich et al., 2007; tom Dieck et al., 2015), adapted for detection of proteins at the cell surface. Cells were incubated in 4 mM L-azidohomoalanine (AHA, Click Chemistry Tools #1066-1000) in the presence or absence of sAPPα or anisomycin. Following incubation, cells were washed (3x quickly) with PBS-MC (pH 7.4), and fixed in PFA in PBS-MCS (pH 7.4). Cell-surface azide-labeled newly synthesized proteins were alkylated with biotin-linked alkyne via a copper-mediated click reaction. Click reaction mixture comprised of 200 μM triazole ligand (Tris ((1-benzyl-1H-1,2,3-triazol-4-yl)methyl) amine; TBTA, Aldrich #678973), 500 μM TCEP (Tris(2-carboxyethyl)phosphine hydro-chloride, Thermo Scientific #PIE-20490), 25 μM Biotin-PEG4-alkyne (Biotin alkyne, Aldrich # B10185) and 200 μM CuSO_4_ in PBS pH 7.8 was incubated on cells overnight at RT. For detection of *de novo* GluA1 and GluA2 proteins cells were permeabilized with 0.5% Triton X-100 in PBS (pH 7.4), and incubated with anti-biotin, anti-GluA1 or anti-GluA2 antibodies diluted in 4% normal goat serum. Donkey anti-mouse PLA^*minus*^, and donkey anti-rabbit PLA^*plus*^ probes were applied, followed by ligation and amplification with Duolink detection reagent Texas Red according to the manufacturer’s instructions. Neuronal somata, dendrites and nuclei were visualized by addition of anti-Guinea Pig Alexa Fluor 488 and DAPI, respectively.

### PLA

The labeling of cell-surface receptor subunit dimers was conducted as per (Lin et al., 2015) and adapted for the detection of AMPARs present at the cell surface. In brief, cells were treated with sAPPα (1 nM) or existing media. Following incubation, cells were washed with PBS-MC (pH 7.4), and fixed in PFA in PBS-MCS (pH 7.4). For experiments investigating GluA1/2-containing AMPARs, using both C-terminal and N-terminal antibodies, cells were incubated in blocking buffer (4% normal goat serum in PBS, 60 min at RT), probed with the N-terminal antibody (90 min, RT), washed (PBS pH 7.4, 3 × 5 min), and subsequently permeabilized with 0.5% Triton X-100 in PBS (15 min, RT). Cells were then probed with the C-terminal antibody in addition to anti-MAP2 for visualization of neuronal structure (90 min, RT) and washed (PBS pH 7.4, 3 × 5 min). For experiments utilizing two N-terminal antibodies, such as those investigating GluA2/3-containing AMPARs, permeabilization was omitted and cells were incubated in blocking buffer (4% normal goat serum in PBS, 60 min at RT) followed by incubation of primary antibodies (90 min, RT). Following this, cells were washed (PBS pH 7.4, 3 × 5 min), permeabilized with 0.5% Triton X-100 in PBS (15 min, RT) and incubated in blocking buffer again (4% normal goat serum in PBS, 60 min at RT) before addition of anti-MAP2 and DAPI for visualization of neuronal structure (90 min, RT). Following addition of antibodies, proximity ligation assay was performed as described above.

### Field Potential Electrophysiology

All experiments conducted on acute tissue were prepared from young adult male Sprague-Dawley rats (42–56 d), as described previously (Mockett et al., 2019). Rats were deeply anesthetized with ketamine (100 mg/kg, i.p.) and decapitated via guillotine. The brains were removed and chilled in ice-cold and oxygenated modified artificial cerebrospinal fluid (aCSF) for which sucrose was substituted for NaCl (composition in mM: sucrose 210, glucose 20, KCl 2.5, NaH_2_PO_4_ 1.25, NaHCO_3_ 26, CaCl_2_ 0.5, MgCl_2_ 3, pH 7.4 when gassed with 95% O_2_-5% CO_2_). Hippocampi were dissected and slices (400 μm) cut using a vibroslicer (Leica, VT1000). Slices were transferred to a porous, transparent membrane in an incubation chamber, and maintained at the interface between air and standard aCSF (in mM: NaCl 124, KCl 3.2, NaH_2_PO_4_ 1.25, NaHCO_3_ 26, CaCl_2_ 2.5, MgCl_2_ 1.3, D-glucose 10, equilibrated with carbogen 95% O_2_-5% CO_2_; 32°C) for 30 min followed by RT for an additional 90 min. Slices were then transferred to the recording chamber containing recirculating aCSF (95% O_2_, 5% CO_2_; 32.5°C), superfused continuously at a rate of 2 mL/min. Baseline field excitatory postsynaptic potentials (fEPSPs) were elicited in area CA1 by stimulation of the Schaffer collateral-commissural pathway at 0.017 Hz (diphasic pulses, 0.1 ms half-wave duration) using a Teflon-coated 50 μm tungsten wire monopolar electrode (A-M Systems Inc., Carlsborg, WA) connected to Grass P511 AC amplifiers via high impedance probes (Grass Instruments Company, West Warwick, United States). Signals were amplified (x1000) with half-amplitude filter cut-offs of 0.3 Hz and 3 kHz. Evoked responses were recorded with a glass microelectrode (A-M systems, 1.0 mm x 0.58 mm, 4”; Catalog No. #601000) filled with aCSF (1.9–2.9 MΩ) and placed in stratum radiatum of area CA1 (approximately 300 μm from the stimulating electrode). During periods of baseline recording the stimulation intensity was adjusted to elicit a fEPSP with an initial slope value of 40% of the maximum elicited when delivering 200 μA of current. Non-saturated LTP was induced by applying a mild theta burst-stimulation protocol (TBS; 5 bursts of 5 pulses at 100 Hz delivered at 200 ms intervals) at baseline stimulus intensity as per Mockett et al. (2019). sAPPα (1 nM) and IEM-1460 (100 μM) were bath-applied by switching to an identical preheated and oxygenated aCSF solution that contained the compound of interest. sAPPα was delivered 30 min prior to TBS and IEM-1460 was delivered 20 min prior to and during the sAPPα administration and continued 10 min post-TBS. Control experiments were routinely interleaved randomly between experimental treatments.

### Microscopy

Images were acquired using an Olympus IX71 inverted light microscope using a 20x/0.45-N.A objective (LUCPFLN) or 4x/0.13-N.A objective (UPFLN). The images were captured using a Hamamatsu Orca-AG camera (C4742-80-12AG) in 1024 × 1024 pixel 8-bit mode and saved as.*tif* files.

### Image Analysis

To quantify the PLA signal a custom-made ImageJ script created by Maximilian Heumüller (Max Planck Institute for Brain Research, Frankfurt) was used (tom Dieck et al., 2015). Following thresholding of the FUNCAT-PLA signal, a ‘MAP2 mask’ was generated capturing the area of the neuron, and the size and intensity of FUNCAT-PLA puncta within the mask were measured and recorded. To analyze the signal within the somatic and dendritic compartments, somata were isolated from each cell as above, and the proximal 50 μm segment all dendrites were straightened using the ‘straighten’ plugin in ImageJ, and analyzed for PLA signal. For the detection of somatic receptor subunit dimers using PLA, analysis included a step to exclude non-specific signal detected within the nucleus using a mask generated by the DAPI channel. For image representation, ImageJ was used to adjust the brightness and contrast equally for all treatment groups. To quantify immunofluorescence, neurons were outlined using ImageJ. An ‘integrated intensity/neuronal area’ value was generated for each cell and somatic compartment, including all dendrites up until intersection with neighboring dendrites. This value was corrected for average background fluorescence by subtracting mean gray values of background fluorescence.

#### Colocalization

To analyze the proximity of PLA signal to the synaptic marker synapsin-1, the ImageJ plugin Just Another Colocalization Plugin (JACoP) was used. In brief, images were separated into single channel images and the proximal 50 μm of MAP2 and PLA signal was isolated. Average PLA and synapsin-1 signal was determined for all treatment groups and applied within the JACoP plugin. In order to determine colocalization, the Mander’s overlap coefficient was used to generate a value proportional to the level of overlap of PLA on synapsin-1 signal, of which a value of -1 indicates negative correlation, 0 indicates no correlation and + 1 indicates a positive correlation. In addition, the proximity of PLA puncta to the synapsin-1 signal was determined by manual measurements of the center of each PLA puncta (determined by the brightest pixel) to the center of mass of the nearest synapsin-1 puncta. Distances defining synaptic (0–2 μm), extrasynaptic (2–4 μm), and non-synaptic (4 + μm) puncta were determined based on the size and observed overlap of the PLA and synapsin-1 puncta (approximately 1 μm; Jarvius et al., 2006; Fantuzzo et al., 2017) at the resolution used.

### Statistical Analysis

#### FUNCAT-PLA & PLA

Prior to data amalgamation, outliers were removed from each experiment using Grubb’s tests, and normality was assessed (D’Agostino & Pearson omnibus normality tests). To examine the expression of newly synthesized proteins and AMPAR complexes at the cell surface using FUNCAT-PLA and PLA, respectively, significance was calculated on amalgamated data per experiment expressed relative to each experimental control by use of one sample *t*-tests (having met the assumptions of independence of observation, homogeneity of variances and approximation to a normal distribution) and F tests to compare variances were applied to datasets when applicable. To examine the effect of sAPPα treatment (1 nM, 30 min) relative to sAPPα (1nM, 2 h) the data were assessed by Mann-Whitney two-tailed U-test. The effect of siRNA was assessed by expressing the normalized sAPPα values + siRNA relative to siRNA alone. To determine whether there was a significant difference between treatment groups and the mean of the controls, data were assessed by use of one sample *t*-tests. To determine whether there was a significant difference between the mean effect of sAPPα alone (fold change relative to control) and the siRNA (sAPPα + siRNA/siRNA), the data were assessed by Mann-Whitney two-tailed U-test. For experiments examining the expression of proteins relative to synaptic puncta, normality was determined by Shapiro-Wilk normality test and significance of the Mander’s overlap coefficient was determined by unpaired *t*-test. For the comparison of synaptic, extrasynaptic, and non-synaptic puncta significance was assessed by two-way ANOVA and Šidák’s multiple comparisons.

#### Immunocytochemistry

Statistics for all immunocytochemistry experiments were performed on raw data using Kruskal-Wallis one-way ANOVA followed by Dunn’s multiple comparisons test. Data were not normally distributed (D’Agostino & Pearson normality test). Outliers within the raw data sets were detected using the Grubb’s test.

#### Electrophysiology

The initial slopes of the fEPSPs were analyzed offline as a measure of synaptic efficacy and expressed as a percentage change from the baseline level. To account for a small but non-significant effect of IEM-1640 on baseline synaptic transmission (−8.3 ± 6.71%, *p* = 0.1127, *n* = 6, one-way ANOVA with Tukey’s multiple comparisons test), the induction of LTP (0–10 min post-TBS, in the presence of IEM-1460) was calculated as the average of the last 10 min of the baseline recording period prior to the application of TBS. Following washout of IEM-1460, responses measuring the persistence of LTP (50–60 min post-TBS) were normalized to the baseline value prior to the addition of IEM-1460. Group means were expressed as the percentage change ± SD. Data acquisition and analysis were performed by custom-built software developed in the Department of Psychology, University of Otago. Normal distribution of data was determined by D’Agostino and Pearson omnibus normality test. Statistical differences between experimental groups was determined by one-way ANOVA followed by Tukey’s multiple comparisons tests at the *p* < 0.05 significance level.

## Results

### sAPPα Promotes the Rapid and Transient Trafficking of *de novo* GluA1-Containing AMPARs to the Somatic and Dendritic Cell Surface

**FIGURE 1:**
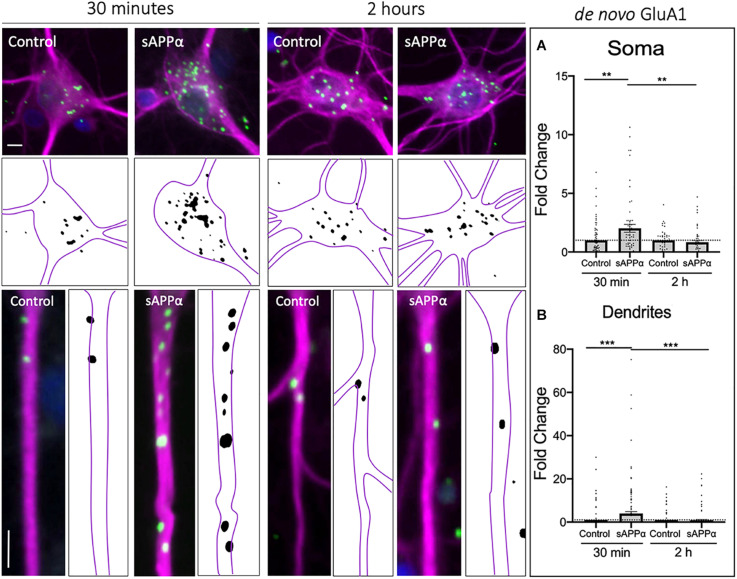
Rapid increase in cell-surface *de novo* GluA1 following treatment. Representative images showing cell-surface *de novo* GluA1 levels in the soma (top panels) and dendrites (lower panels) from 30 min (left) and 2 h (right) control and sAPPα-treated conditions. **(A)** Average data showing 1 nM sAPPα promotes an increase in the soma (*n* = 50–71 cells) following 30 min but not 2 h. **(B)** Average data showing 1 nM sAPPα (30 min) promotes an increase in *de novo* GluA1 at the dendritic cell surface follow 30 min but not 2 h treatments (*n* = 104–128). Outliers were removed from each experiment prior to amalgamation using Grubb’s tests, and normality was detected by D’Agostino and Pearson omnibus normality tests. All data are expressed relative to control, as mean ± SEM from 3 experiments. Significance relative to control was assessed by student’s *t*-test, significance between sAPPα-treated groups was assessed by Mann-Whitney two-tailed U-test. **p ≤ 0.01, ***p ≤ 0.001. Representative images show neuronal soma (upper panels) and dendrites (lower panels; MAP2, 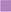; GluA1, 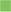; DAPI, 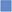). Scale bars = 10 μm.

It is not known whether sAPPα induces the rapid synthesis of GluA1-containing AMPARs or whether these *de novo* molecules are trafficked to the cell surface and thus contribute to plasticity. Thus, to examine specifically the cell-surface population of *de novo* GluA1, we utilized FUNCAT-PLA under detergent-free conditions. We found that sAPPα treatment (1 nM; 30 min) significantly increased the cell-surface expression of *de novo* GluA1 in both the soma (2.01 ± 0.33 mean ± SEM, *p* = 0.0039; Figure 1A) and dendrites (3.97 ± 0.32, *p* = 0.0009; Figure 1B) of cultured hippocampal neurons. Interestingly, following 2 h of sAPPα treatment, there was no detectable change in *de novo* somatic (0.83 ± 0.15, *p* = 0.265; Figure 1A) or dendritic (0.86 ± 0.29, *p* = 0.673; Figure 1B) GluA1. Indeed, cell-surface expression of GluA1 at 2 h was significantly decreased relative to 30 min of sAPPα treatment on both the soma (*p* = 0.0058) and dendrites (*p* = 0.0005). Interestingly, in parallel experiments using immunocytochemistry with non-permeabilized conditions to allow detection of both extant and newly made cell surface GluA1, we were able to detect increases in GluA1 in the dendrites. We observed a modest increase by 30 min (2.37 ± 0.37, *p* = 0.0787), which reached statistical significance by 2 h (3.00 ± 0.44, *p* = 0.0002) and remained significantly elevated at 4 h (2.50 ± 0.39, *p* = 0.0204). We found no changes in the soma at any time point (30 min: 1.09 ± 0.12, *p* = 0.8998; 2 h: 1.066 ± 0.82, *p* = 0.2694, 4 h: 1.043 ± 0.15, *p* = 0.3191; see Supplementary Figure 1). Together, these results suggest that sAPPα induces the rapid *de novo* synthesis of GluA1 subunits, which are trafficked to the cell surface by 30 min. These newly made subunits are later internalized within 2 h of treatment, yet levels of extant GluA1-containing receptors remain elevated for up to 4 h.

### Rapid Decrease in Dendritic *de novo* Cell Surface GluA2 Following sAPPα Treatment

**FIGURE 2:**
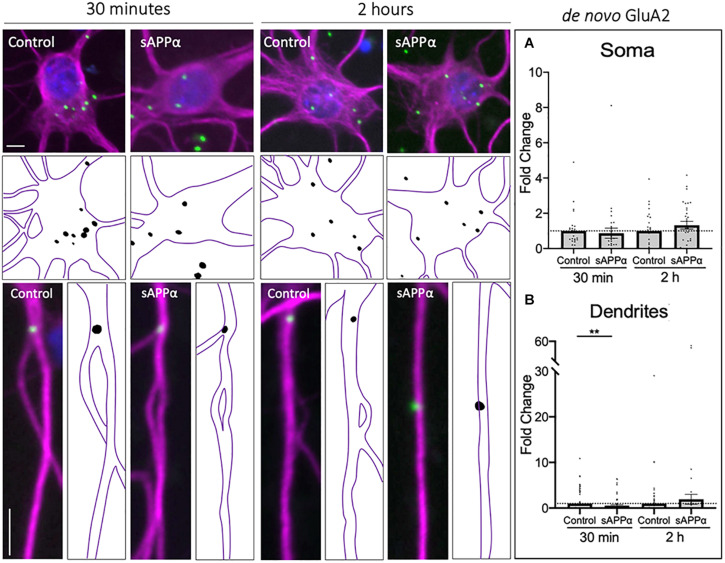
Rapid decrease in *de novo* cell-surface GluA2 following treatment. Representative images showing cell-surface *de novo* GluA2 levels in the soma (top panels) and dendrites (lower panels) from 30 min (left) and 2 h (right) controls and sAPPα treated conditions. **(A)** Average data showing 1 nM sAPPα does not affect somatic (*n* = 25–31 cells) *de novo* GluA2. **(B)** Average data showing 1 nM sAPPα (30 min) significantly decreases dendritic *de novo* GluA2 (*n* = 71–76). All data are expressed relative to control, as mean ± SEM from 3 experiments. Outliers were removed from each experiment prior to amalgamation using Grubb’s tests, and normality was detected by D’Agostino and Pearson omnibus normality tests. Significance was assessed by student’s *t*-test, **p ≤ 0.01. Representative images show neuronal soma (upper panels) and dendrites (lower panels; MAP2; 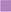; GluA1, 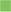; DAPI; 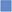). Scale bars = 10 μm.

As the GluA1 subunit is a constituent of either GluA1/2 or GluA1-homomeric receptors, to determine the likely composition of *de novo* AMPARs formed following sAPPα treatment, we next examined the temporal expression of *de novo* GluA2 at the cell surface, in both the somatic and dendritic compartments. Here, we found that *de novo* cell-surface GluA2 remained unaffected at the soma by either 30 min (0.86 ± 0.28, *p* = 0.654) or 2 h (1.32 ± 0.21, *p* = 0.148; Figure 2A) sAPPα treatments. Conversely, there was an early decrease of *de novo* cell-surface GluA2 at the dendritic cell surface (30 min: 0.54 ± 0.17, *p* = 0.009), which returned to control levels within 2 h (1.92 ± 1.08, *p* = 0.395; Figure 2B). From this, we can infer that while sAPPα upregulates the expression of *de novo* GluA1, it inhibits the synthesis or otherwise restricts expression of *de novo* GluA2 at the cell surface. Together, these results indicate that sAPPα acts both to upregulate cell surface *de novo* GluA1-containing homomeric AMPARs and downregulate *de novo* GluA2-containing AMPARs.

### sAPPα Enhances the Extrasynaptic but Not the Synaptic Population of *de novo* Cell Surface GluA1

**FIGURE 3:**
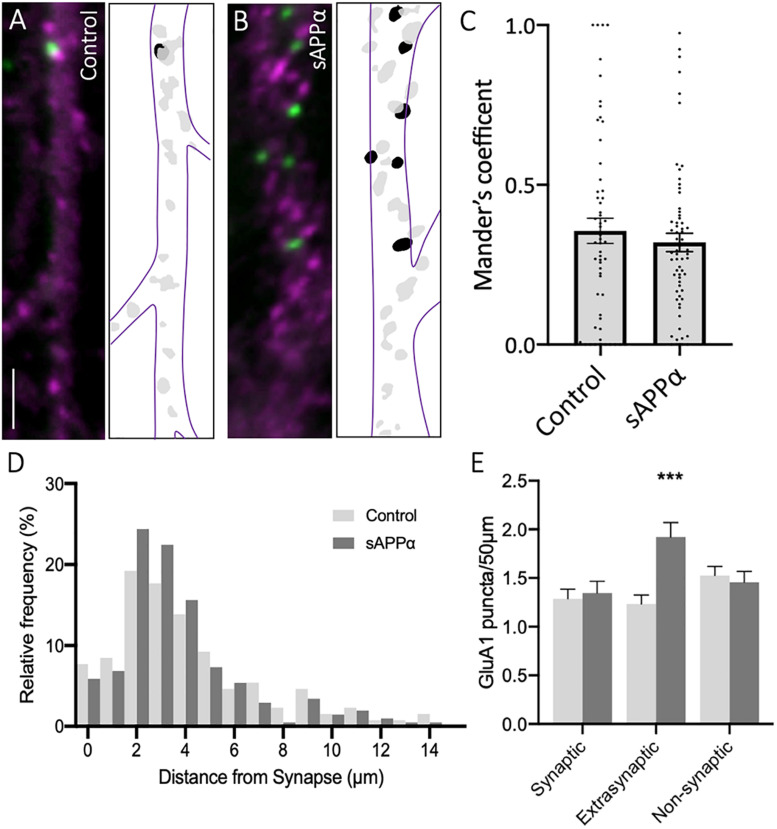
Secreted amyloid precursor protein-alpha enhances *de novo* GluA1 at the extrasynaptic membrane. Representative images showing *de novo* cell-surface GluA1 in **(A)** control and **(B)** sAPPα-treated conditions. Representative images show dendrites (50 μm; synapsin-1, 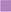; GluA1, 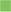). Scale bar = 10 μm. **(C)** No significant difference was observed in the Mander’s overlap coefficient following sAPPα-treatment (1 nM, 30 min; *n* = 20 cells, 58–65 dendrites). Significance was assessed by Mann-Whitney two-tailed U-test. **(D)** Frequency histogram of the distribution of *de novo* GluA1 puncta in relevance to synapsin-1 immunofluorescence. Distances were calculated for individual PLA puncta with respect to the closest synapsin-1 center of mass. Synaptic PLA showed overlapping centers within 0–2 μm, puncta within 2–4 μm were considered extrasynaptic, and puncta beyond 4 μm were considered non-synaptic (*n* = 58–65 dendrites, 130–205 puncta). **(E)** Quantification of GluA1 puncta abundance at the synaptic, extrasynaptic, and non-synaptic membrane (*n* = 58–65 dendrites, 130–205 puncta). Normality was determined by Shapiro-Wilk normality test. All data are expressed relative to control, as mean ± SEM from 3 experiments. Significance was assessed by two-way ANOVA and Šidák’s multiple comparisons, ***p ≤ 0.0005.

To further explore the localization of *de novo* GluA1 subunits, we determined the proportion of dendritic GluA1-containing AMPARs, identified by FUNCAT-PLA, associated with the presynaptic marker synapsin-1 (Figures 3A,B). Synaptic overlap of GluA1 puncta was determined using Mander’s overlap coefficient (MOC, Figure 3C). We found no significant difference between control and sAPPα-treated (1 nM, 30 min) conditions (control MOC: 0.35 ± 0.03 mean ± SEM, sAPPα: 0.31 ± 0.02; *p* = 0.7038; Figure 3C), indicating that 30 min sAPPα treatment did not increase the proportion of *de novo* synaptic GluA1. We next determined the proportion of synaptic, extrasynaptic and non-synaptic cell surface *de novo* GluA1 puncta relative to proximity to synapsin-1-positive synapses. We defined synaptic, extrasynaptic, and non-synaptic PLA puncta as 0–2, 2–4, and > 4 μm from the closest synapsin-1 center of mass, ‘. This analysis showed a shift in the frequency of *de novo* GluA1 puncta present at synapses following sAPPα treatment, increasing the proportion of GluA1 puncta 2–4 μm proximal to the synapse (Figure 3D). In line with this, we found a significant increase in the number of *de novo* GluA1 puncta at extrasynaptic sites (control: 1.23 ± 0.09 puncta; sAPPα: 1.92 ± 0.14; *p* = 0.0003). No change was detected at synaptic (control: 1.28 ± 0.10; sAPPα: 1.34 ± 0.12; *p* = 0.9872), or non-synaptic (control: 1.52 ± 0.09; sAPPα: 1.45 ± 0.11; *p* = 0.9655) sites (Figure 3E). These results suggest that sAPPα rapidly enhances the extrasynaptic pool of *de novo* GluA1-containing AMPARs within the dendrites.

### Internalization of *de novo* GluA1 Is Dependent on Arc Expression

**FIGURE 4:**
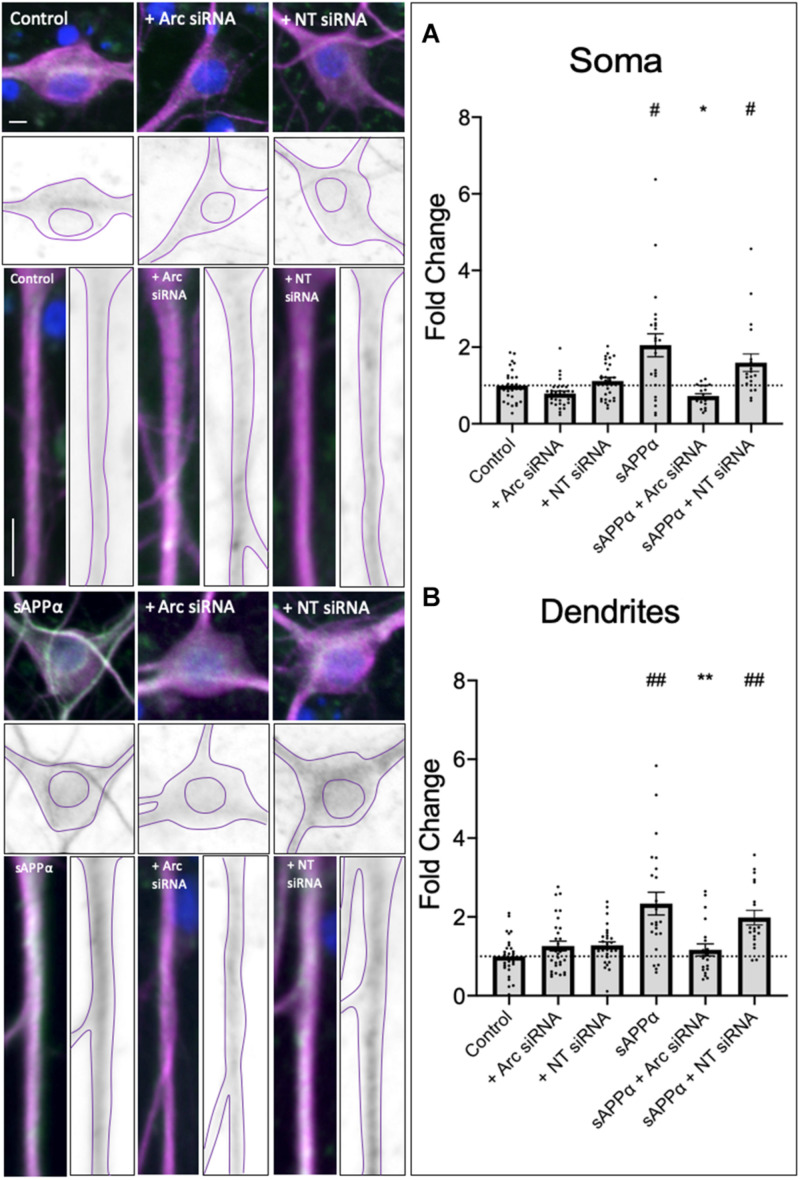
Secreted amyloid precursor protein-alpha promoted Arc expression is affected by Accell^TM^ Arc siRNA. Representative images show Arc protein levels the soma (upper panels) and dendrites (lower panels) in control and sAPPα-treated primary hippocampal neurons ± co-treatment with Arc siRNA or non-targeted (NT) control siRNA. In both the **(A)** soma and **(B)** dendrites (*n* = 19–30), sAPPα treatment significantly enhanced Arc expression in sAPPα-only and sAPPα + NT siRNA conditions. This effect was inhibited by co-treatment with the Arc siRNA. Outliers were removed from each experiment prior to amalgamation using Grubb’s tests, and normality was detected by D’Agostino and Pearson omnibus normality tests. Significance was calculated using a Kruskal–Wallis one-way ANOVA with Dunn’s multiple comparisons test on raw data, and expressed as fold change relative to the experimental control. All data are expressed relative to control, as mean ± SEM from 3 experiments. Hashes (#) denote significant difference from control, asterisks (*) denote significant difference from sAPPα-treated. ^#^/*p ≤ 0.05, **/^##^*p* ≤ 0.005. Representative images show neuronal soma, dendrites (MAP2, 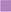), Arc protein (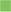), nuclei (DAPI, 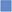). Scale bars = 10 μm.

Prolonged treatment of sAPPα (1 nM, 2 h) enhances the *de novo* transcription and translation of the immediate early gene Arc, a protein intrinsically linked to glutamate receptor expression at the cell surface (Livingstone et al., 2019). Treatment of primary hippocampal cultures with an siRNA (1 μM, pre-treatment: 60 min, treatment: 2 h) targeting Arc mRNA or a non-targeting (NT) siRNA with no known homology to rat or human genes had no significant effect on basal Arc protein expression in either the soma (Arc siRNA: 0.78 ± 0.06, *p* ≥ 0.99; NT siRNA: 1.11 ± 0.09, *p* = 0.0509; Figure 4A) or the dendrites (Arc siRNA: 1.26 ± 0.12, *p* = 0.8774; NT siRNA: 1.28 ± 0.08, *p* = 0.7561; Figure 4B). As previously shown (Livingstone et al., 2019), sAPPα treatment (1 nM, 2 h) enhanced both somatic (2.05 ± SEM, *p* = 0.0306; Figure 4A) and dendritic (2.33 ± 0.29, *p* = 0.0010; Figure 4B) Arc protein expression. Co-treatment with sAPPα and the NT siRNA (1 μM, pre-treatment: 60 min, co-treatment: 2 h) also enhanced Arc expression (soma: 1.59 ± 0.23, *p* = 0.0198; dendrites: 1.98 ± 0.18, *p* = 0.0022), and no significant difference was detected between sAPPα-treated and sAPPα + NT siRNA in either the soma or dendrites (*p* ≥ 0.99). Thus, NT siRNA does not affect the expression of Arc protein. By contrast, co-treatment with sAPPα and the Arc siRNA significantly reduced Arc protein expression in both the soma (0.72 ± 0.27, *p* = 0.0100) and dendrites (1.16 ± 0.68, *p* = 0.0066) relative to sAPPα treatment alone, and was not significantly different from control in either compartment (*p* ≥ 0.99). Together, these results indicate that Arc siRNA effectively inhibits sAPPα-induced *de novo* Arc synthesis in our primary hippocampal cultures.

**FIGURE 5:**
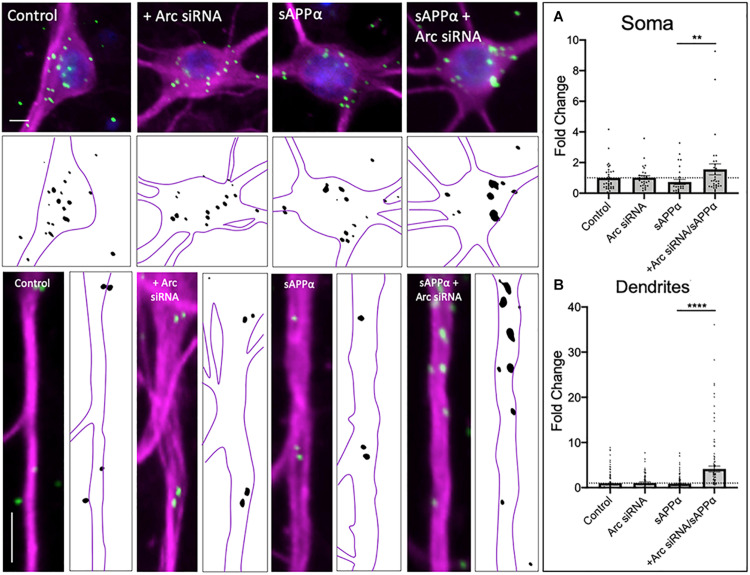
*de novo* GluA1 persist at the cell surface following siRNA-mediated knockdown of Arc protein. Representative images of somatic (upper panels) and dendritic (lower panels) of control and sAPPα-treated conditions ± treatment with the Arc siRNA. **(A)** Average data showing treatment with sAPPα (1 nM, 2 h) and Arc siRNA (1 μM, pre-treatment: 60 min, co-treatment: 2 h) promotes an increase in **(A)** somatic (*n* = 28–35 cells) and **(B)** dendritic (*n* = 79–132) *de novo* cell-surface GluA1. Outliers were removed from each experiment prior to amalgamation using Grubb’s tests, and normality was detected by D’Agostino and Pearson omnibus normality tests. All data are expressed relative to control, as mean ± SEM from 3 experiments. Data assessing the effect of siRNA are expressed as mean ratio of sAPPα + Arc siRNA/Arc siRNA alone ± SEM. Significance between control and treatment was assessed by student’s *t*-test, significance between sAPPα-treated and sAPPα + siRNA-treated was assessed by Mann-Whitney two-tailed U-test, **p ≤ 0.001, ****p ≤ 0.0001. Representative images show neuronal soma (upper panels) and dendrites (lower panels; MAP2, 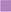; GluA1, 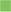; DAPI, 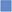). Scale bars = 10 μm.

As *de novo* GluA1 levels at the cell surface did not persist following prolonged (2 h) treatment of sAPPα, we next employed siRNA knockdown of *de novo* Arc protein to assess whether inhibition of Arc protein synthesis affects *de novo* GluA1 cell surface levels. As expected, sAPPα treatment for 2 h resulted in no change in either somatic (0.73 ± 0.16, *p* = 0.118) or dendritic (0.85 ± 0.85, *p* = 0.374) expression of *de novo* cell surface GluA1, relative to control conditions (Figures 4A,B). However, in the presence of Arc siRNA (preincubation: 1 μM, 60 min, followed by co-incubation with sAPPα: 1 nM, 2 h), we observed a significant increase in the expression of *de novo* GluA1 at both the somatic (1.55 ± 0.36, *p* = 0.0043; Figure 5A) and dendritic (4.14 ± 0.65, *p* ≤ 0.0001; Figure 5B) cell surface, relative to sAPPα-only conditions. Finally, treatment of cultures with the NT siRNA alone had no observable effect on the levels of *de novo* cell surface GluA1 at the soma (0.94 ± 0.15, *p* = 0.7247, *n* = 23; Supplementary Figure 2A), or dendrites (1.08 ± 0.21, *p* = 0.7050, *n* = 89; Supplementary Figure 2B) relative to control, nor relative to NT + sAPPα treatment in the soma (0.69 ± 0.12, *p* = 0.2790; *n* = 18; Supplementary Figure 2A) or dendrites (0.95 ± 0.20, *p* = 0.2077, *n* = 98; Supplementary Figure 2B), indicating that the observed effects are due to the specific to the addition of Arc siRNA and subsequent knockdown of Arc protein expression and not as a result of non-specific siRNA-mediated effects. Therefore, these results show that following the rapid expression of *de novo* GluA1-containing AMPARs at the cell surface, these AMPARs are subsequently endocytosed in an Arc-dependent manner.

### sAPPα Promotes the Delayed Expression of Cell Surface GluA1/2-Containing AMPARs

In response to synaptic activity (Bagal et al., 2005; Kopec et al., 2006; Guire et al., 2008; Tanaka and Hirano, 2012; Park et al., 2016; Pandya et al., 2018), behavioral learning (Whitlock et al., 2006; Fachim et al., 2016), and neuromodulators (Leonoudakis et al., 2008; Jourdi and Kabbaj, 2013), cell surface or synaptic accumulation of GluA2-containing AMPARs has been shown to occur, typically following the incorporation of GluA1-containing homomers (Shi et al., 1999; Bellone and Lüscher, 2005, 2006; Guire et al., 2008; Hong et al., 2013; Park et al., 2016).

**FIGURE 6:**
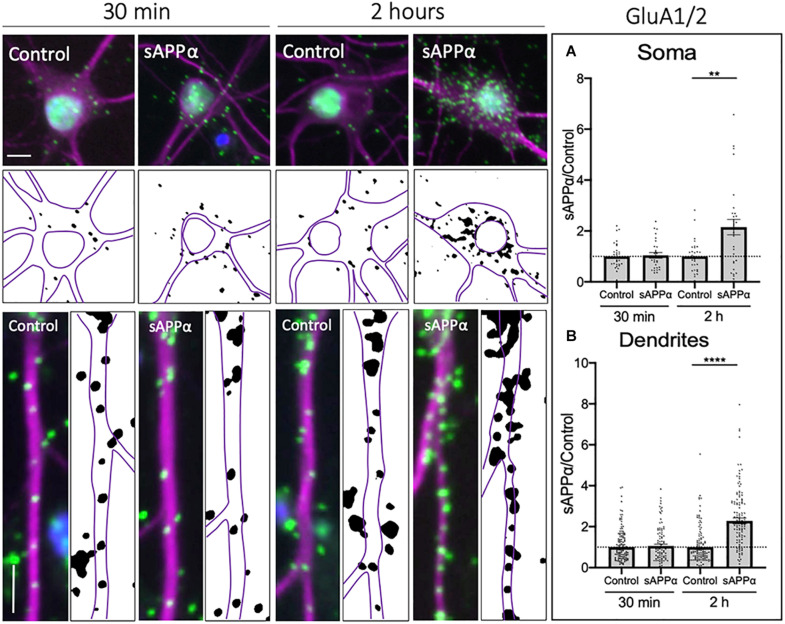
Secreted amyloid precursor protein-alpha enhances cell-surface GluA1/2-containing AMPARs. Representative images showing cell-surface GluA1/2 levels in the soma (top panels) and dendrites (lower panels) from 30 min (left) and 2 h (right) controls and sAPPα treated conditions. Average data showing 1 nM sAPPα promotes an increase in the **(A)** soma (*n* = 28–31 cells) and **(B)** dendrites (*n* = 98–142) following 2 h- but not 30 min treatments. All data are expressed relative to control, as mean ± SEM from 3 experiments. Normality was detected by D’Agostino and Pearson omnibus normality tests and significance was assessed by student’s *t*-test, **p ≤ 0.001, ****p ≤ 0.0001. Representative images show neuronal soma (upper panels) and dendrites (lower panels; MAP2, 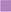; GluA1/2, 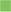; DAPI, 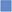). Scale bars = 10 μm.

While previous research examining acute sAPPα treatments (Mockett et al., 2019) found no evidence for an increase in *de novo* GluA2 synthesis in response to sAPPα, this does not exclude the possibility that sAPPα additionally promotes the trafficking of pre-existing GluA2-containing AMPARs to the cell surface. Therefore, we extended our analysis to examine cell surface populations of GluA1- and GluA2-containing (GluA1/2) AMPARs within somatic and dendritic compartments. Here, we utilized PLA to detect the coincident proximity of GluA1 and GluA2 AMPAR complexes at the cell surface. We found no significant increase in GluA1/2-containing AMPARs at the cell surface following a 30 min sAPPα treatment, in either the soma (1.04 ± 0.11, *p* = 0.709; Figure 6A) or dendrites (1.05 ± 0.09, *p* = 0.54; Figure 6B) of cultured primary hippocampal neurons. However, the cell surface expression of GluA1/2 AMPARs significantly increased in both the soma (2.15 ± 0.30, *p* = 0.0013; Figure 6A), and dendrites (2.28 ± 0.14, *p* ≤ 0.0001; Figure 6B) by 2 h. These results indicate that GluA1/2 AMPARs are expressed at the cell surface following prolonged sAPPα treatment.

### GluA1/2-Containing AMPARs Localize to Synapses

**FIGURE 7:**
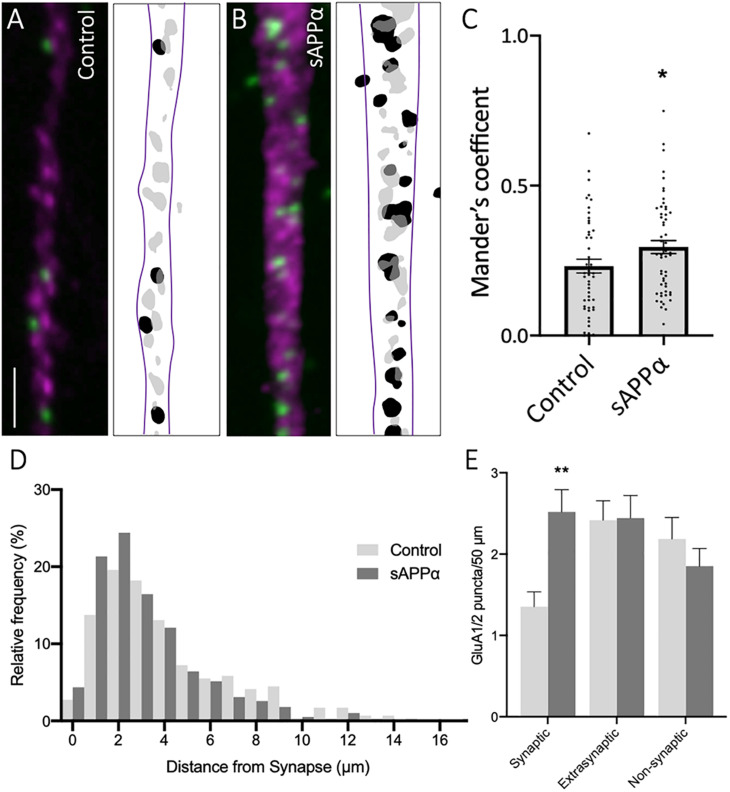
Secreted amyloid precursor protein-alpha enhances GluA1/2 AMPARs at synapses. Representative images showing cell-surface GluA1/2 AMPARs in **(A)** control and **(B)** sAPPα-treated conditions Representative images show dendrites (50 μm; synapsin-1, 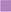; GluA1/2, 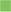). Scale bar = 10 μm. **(C)** sAPPα treatment (1 nM, 2 h) showed a significant increase in Mander’s overlap coefficient (*n* = 20 cells, 49–54 dendrites). Significance was assessed by unpaired *t*-test. **(D)** Frequency histogram of the distribution of GluA1/2 puncta in relevance to synapsin-1 immunofluorescence. Distances were calculated for individual PLA puncta with respect to the closest synapsin-1 center of mass. Synaptic PLA showed overlapping centers within 0–2 μm, puncta within 2–4 μm were considered extrasynaptic, and puncta beyond 4 μm were considered non-synaptic (*n* = 49–54 dendrites, 291–389 puncta). **(E)** Quantification of GluA1/2 puncta abundance at the synaptic, extrasynaptic, and non-synaptic membrane (*n* = 49–54 dendrites, 291–389 puncta). Normality was determined by Shapiro–Wilk normality test. All data are expressed relative to control, as mean ± SEM from 3 experiments. Significance was assessed by two-way ANOVA and Šidák’s multiple comparisons, *p = 0.0467, **p ≤ 0.0029.

We further sought to determine the synaptic localization of cell surface GluA1/2 AMPARs (Figures 7A,B). Synaptic overlap of GluA1/2 puncta was determined using MOC (Figure 7C). Here, we found a significant increase in MOC in sAPPα-treated (1 nM, 2 h) conditions (MOC: 0.295 ± 0.02, *p* = 0.0467, Figure 7C), relative to control (control: 0.231 ± 0.02; Figure 7C), indicating that sAPPα treatment increased the proportion of GluA1/2 AMPARs at synaptic sites. To validate this finding, we determined the proximity of synaptic, extrasynaptic, and non-synaptic cell surface GluA1/2 puncta relative to the proximity of synapsin-1-positive synapses, as above (Figure 7). Supporting the observed increase in MOC, we observed a shift in the frequency ofnd remained compara GluA1/2 AMPARs present at synapses following sAPPα treatment, increasing the proportion of GluA1 puncta 0–1 μm proximal to the synapse (Figure 7D). Expanding on this, we found a significant increase in the number of GluA1/2 at synaptic sites (control: 1.35 ± 0.018 puncta; sAPPα: 2.51 ± 0.27, *p* = 0.0029), however, no change was detected at extrasynaptic (control: 2.41 ± 0.24; sAPPα: 2.44 ± 0.27, *p* = 0.9997), or non-synaptic (2.18 ± 0.26; sAPPα: 1.85 ± 0.21, *p* = 0.7089) sites (Figure 7E). These results show that sAPPα enhances the synaptic pool of GluA1/2 AMPARs within the dendrites of primary hippocampal neurons, potentially replacing *de novo* GluA1 homomers.

### sAPPα Promotes the Rapid and Sustained Internalization of GluA2/3-Containing AMPARs

GluA2- and GluA3-containing (GluA2/3) AMPARs comprise the second largest subtype of hippocampal AMPARs. The presence of GluA2/3 AMPARs is thought to denote synaptic maturity, with expression increasing throughout development and acting to replace GluA1/2 AMPARs at the synapse via constituent recycling (Zhu et al., 2000; Shinohara and Hirase, 2009). Interestingly, previous research has shown that GluA3-containing AMPARs are regulated by *in vivo* LTP (Williams et al., 2007), and *in vitro* LTD (Holman et al., 2007), and growth factor treatment (Narisawa-Saito et al., 1999). Curiously, GluA3-containing AMPARs do not appear to directly regulate the expression of LTP or context fear memory formation (Meng et al., 2003; Humeau et al., 2007), but their removal from the synapse may be an essential step required for LTD (Holman et al., 2007). Regardless, their synaptic expression is considered essential for basal synaptic transmission (Meng et al., 2003).

**FIGURE 8:**
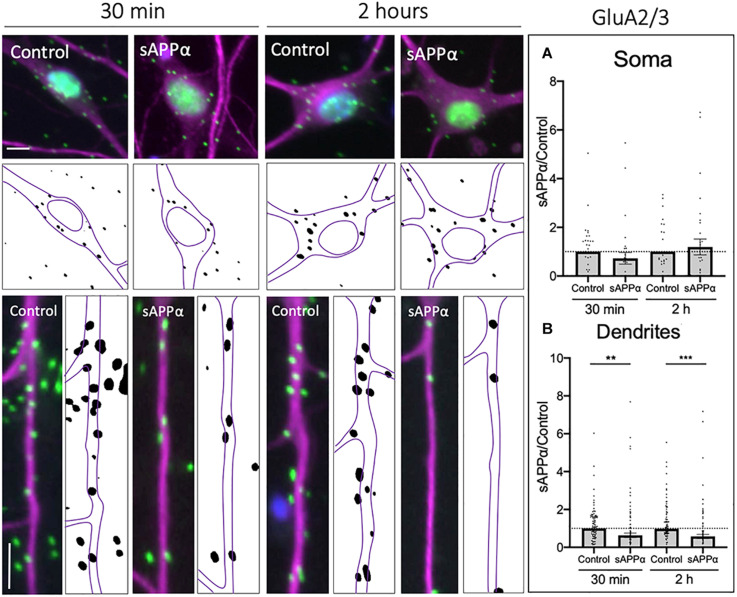
Secreted amyloid precursor protein-alpha downregulates cell-surface GluA2/3-containing AMPAR expression. Representative images showing cell-surface GluA2/3 levels in the soma (top panels) and dendrites (lower panels) from 30 min (left) and 2 h (right) controls and sAPPα treated conditions. **(A)** Average data showing sAPPα (1 nM) does not affect somatic GluA2/3 following 30- and 2 h treatments (*n* = 30–32 cells). **(B)** Average data showing sAPPα (1 nM; 30 min, 2 h) promotes a decrease in the dendrites (*n* = 109–111). All data are expressed relative to control, as mean ± SEM from 3 experiments. Normality was detected by D’Agostino and Pearson omnibus normality tests and significance was assessed by student’s *t*-test, **p = 0.0033, ***p = 0.0002. Representative images show neuronal soma (upper panels) and dendrites (lower panels; MAP2, 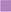, GluA2/3, 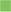; DAPI, 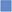). Scale bars = 10 μm.

Using PLA to label cell surface GluA2/3-containing AMPARs, we found that sAPPα (1 nM, 30 min) significantly decreased the cell surface expression of GluA2/3-containing AMPARs within the dendrites of cultured neurons (0.63 ± 0.12, *p* = 0.0033; Figure 8B), which remained decreased at 2 h (0.57 ± 0.11, *p* = 0.0002; Figure 8B). Interestingly, somatic levels of GluA2/3 AMPARs remained unaffected following both 30 min (0.72 ± 0.23, *p* = 0.255; Figure 8A), and 2 h (1.19 ± 0.32, *p* = 0.557) treatments. These results may indicate that GluA3-containing AMPARs are removed from the dendritic cell surface to permit the insertion of GluA1 homomers or GluA1/2- AMPARs (Shi et al., 2001). Alternatively, the removal of GluA3-containing AMPARs may reflect homeostatic processes, maintaining synaptic activity within a physiological range (Rial Verde et al., 2006; Diering et al., 2014; Tan et al., 2015).

### CP-AMPARs Contribute to the Initial Enhancement of sAPPα-Enhanced LTP

**FIGURE 9:**
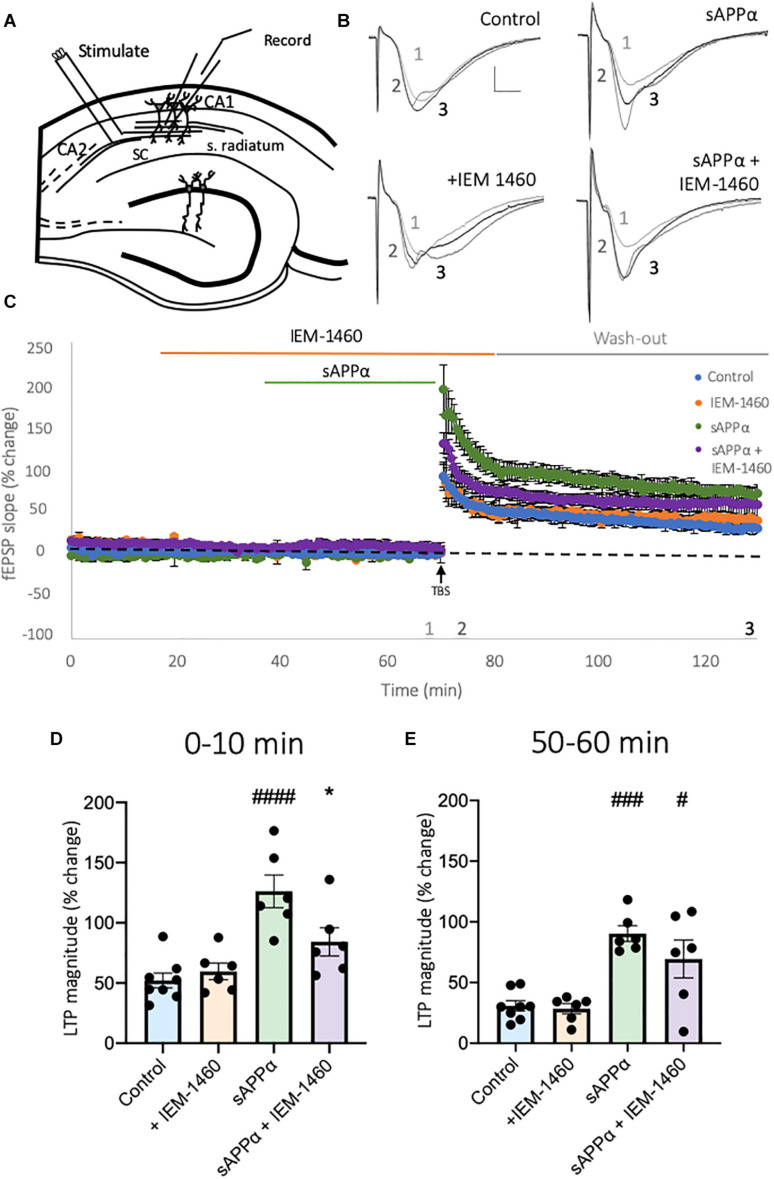
Secreted amyloid precursor protein-alpha-enhanced LTP induction is dependent on activation of CP-AMPARs. **(A)** Representative schematic of transverse hippocampal slices showing the positioning of stimulation and recording electrodes in area CA1. **(B)** Representative fEPSP traces taken at the end of baseline recording (1), upon TBS (2), and 60 min post-TBS (3). Scale bar = 1 mV, 5 ms. **(C)** Average traces of control slices, receiving only a mild TBS (5 bursts at 5 Hz, 5 pulses per 100 Hz/burst; 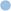, *n* = 8 slices), IEM-1460 (100 μM; 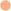, *n* = 6 slices), sAPPα (1 nM; 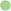, *n* = 6 slices) and sAPPα + IEM-1460-treated (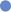, *n* = 6 slices) conditions. All conditions are normalized to the average of baseline 10 min before TBS, and all data are presented as mean% change ± SD. Data shows summary histograms for **(D)** early and **(E)** late potentiation following TBS. To account for the minor effect of IEM-1460 on baseline synaptic transmission, data examining early potentiation in the presence of IEM-1460 are averaged from the first **(D)** 10 min post-TBS for each experimental group relative to baseline, averaged 10 min before TBS. Following washout late potentiation was measure from the final **(E)** 10 min post-TBS relative to baseline, averaged 10 min before application of IEM-1460. *p = 0.0334, ^#^*p* = 0.0149,^ ###^*p* = 0.0002, ^####^*p* ≤ 0.0001. Normality was detected by Shapiro-Wilk normality test. Significance was assessed by one-way ANOVA followed by Tukey’s multiple comparisons tests. Hashes (#) indicate significance between control and sAPPα-treated; asterisks (*) indicate significance between sAPPα- and sAPPα + IEM-1460–treated. SC = Schaffer collaterals, s. radiatum = Stratum radiatum.

The work described above indicates that sAPPα enhances the extrasynaptic expression of *de novo* GluA1, but not GluA2-containing AMPARs in primary hippocampal neurons (Figures 1, 2). To determine whether these GluA1-containing AMPARs comprise functional homomeric Ca^2+^-permeable receptors, we assessed their involvement in plasticity in acute hippocampal slices, building on Mockett et al. (2019) and Richter et al. (2018) who have previously shown that sAPPα enhances submaximal LTP in area CA1. We first examined the dependence of CP-AMPARs during TBS-induced LTP alone (Figures 9A–C), by utilizing the CP-AMPAR antagonist IEM-1460. Following TBS, continued perfusion of IEM-1460 (100 μM; 10 min) had no significant effect on the induction of LTP (0–10 min post-TBS; control: 52.10 ± 6.19% of baseline, mean ± SD; IEM-1460: 59.66 ± 6.94% of baseline, *p* = 0.9399; Figures 9C,D), and remained comparable to no-drug control slices following washout (50–60 min post-TBS; control: 26.64 ± 4.33% of baseline; IEM-1460: 27.99 ± 4.12% of baseline, *p* = 0.9969; Figures 9C,E), supporting past research (Adesnik and Nicoll, 2007; Gray et al., 2007; Asrar et al., 2009). Due to this, we conclude that CP-AMPARs do not play a significant role in the potentiation of synaptic transmission following a mild TBS protocol, and therefore conclusions drawn following treatment of slices with sAPPα may be attributed to mechanisms activated by sAPPα alone.

We next examined whether the enhancement of LTP induction following sAPPα treatment (1 nM, 30 min) was dependent on the function of CP-AMPARs. Here, slices were perfused with IEM-1460 (100 μM, 50 min) before TBS, and 10 min post-TBS stimulation. Slices were also perfused with sAPPα 30 min before TBS in the presence or absence of IEM-1460. As shown in Figure 9, relative to control slices, pre-treatment with sAPPα (1 nM, 30 min) enhanced both the induction (126.2 ± 13.55% of baseline, *p* ≤ 0.0001; Figures 9C,D) and persistence (77.41 ± 10.66% of baseline, *p* = 0.0002; Figures 9C,E) of LTP, confirming previously observed enhancements described by Mockett et al. (2019). Co-application of IEM-1460 significantly inhibited the sAPPα-enhanced early potentiation (84.19 ± 11.74% of baseline, *p* = 0.0334; Figures 9C,D), while wash-out of IEM-1460 resulted in the recovery of potentiation, which was no longer different from sAPPα treatment alone (70.78 ± 15.44% of baseline, *p* = 0.3518; Figures 9C,E), but was significantly different from control slices (*p* = 0.0149). These results indicate that at least the initial period of the sAPPα-mediated enhancement of LTP is due to the rapid incorporation of functional CP-AMPARs into the synapse.

## Discussion

Secreted amyloid precursor protein-alpha plays a crucial role in synaptic plasticity and memory. Knockdown of APP or inhibition of sAPPα significantly reduces LTP in the dentate gyrus and impairs spatial memory and inhibitory avoidance in rats and passive avoidance in chicks (Mileusnic et al., 2000; Taylor et al., 2008). Similarly, inhibition of α-secretase activity impairs hippocampal LTP and spatial memory (Taylor et al., 2008). Importantly, these deficits are restored by either acute administration of sAPPα or peptide derivatives (Taylor et al., 2008; Morrissey et al., 2019) or by genetic overexpression (Ring et al., 2007; Tan et al., 2018). Understanding how sAPPα orchestrates these effects is a critical step if the administration of sAPPα or derivatives is to be considered as a therapeutic strategy in neurodegenerative diseases. Mounting evidence suggests that sAPPα regulates LTP and memory through the regulation of new gene transcription, protein synthesis, and glutamate receptor trafficking (Richter et al., 2018; Mockett et al., 2019). In the current experiments, we have shown that sAPPα differentially regulates the synthesis and cell surface expression of AMPARs in hippocampal neurons. Importantly, this includes the rapid synthesis and trafficking of *de novo* GluA1 homomeric AMPARs, crucial to the sAPPα-driven enhancement of LTP.

### Regulated Synaptic Expression of CP-AMPARs

Secreted amyloid precursor protein-alpha enhances the synthesis of GluA1 but not GluA2 subunits (Mockett et al., 2019). Although sAPPα alters transcription profiles (Stein et al., 2004; Ryan et al., 2013), it has not been shown to alter levels of AMPAR subunit mRNA within a 2 h treatment window. sAPPα does, however, enhance protein synthesis within isolated hippocampal synapses (Claasen et al., 2009), and it is well established that AMPAR subunits, including GluA1, undergo synthesis within dendrites (Torre and Steward, 1992; Kacharmina et al., 2000; Grooms et al., 2006; Sutton and Schuman, 2006) for direct exocytosis at extrasynaptic sites (Choquet, 2018). Thus, it is likely that the rapid *de novo* synthesis of GluA1 results from translation of pre-existing mRNA. It is yet to be determined whether sAPPα-induced GluA1 synthesis occurs within dendrites or is delivered to dendrites via lateral diffusion from somatic sites (Borgdorff and Choquet, 2002; Adesnik et al., 2005) or active transport throughout the dendrites (Passafaro et al., 2001; Hangen et al., 2018).

### Role of *de novo* CP-AMPARs in sAPPα-Enhanced LTP

We have shown that *de novo* GluA1 is trafficked to extrasynaptic sites and is later internalized, but not degraded, as intracellular levels remain elevated (Mockett et al., 2019). As extrasynaptic AMPARs are necessary for the induction of LTP (Oh et al., 2006; Penn et al., 2017) and increasing the proportion of extrasynaptic AMPARs enhances the expression of LTP (Oh et al., 2006), the enhanced GluA1 expression likely provides reservoirs of CP-AMPARs which could underpin the sAPPα-mediated priming of LTP. Indeed, we confirmed that sAPPα does not affect responses to baseline stimulation (Richter et al., 2018; Mockett et al., 2019), nor did IEM-1460 affect baseline responses in sAPPα-treated slices any more than control slices, all supporting the view that the enhancement of cell-surface GluA1 expression by sAPPα is strictly extrasynaptic. However, pre-treatment with sAPPα did enhance LTP induction in a manner that was dependent on CP-AMPARs, likely due to lateral diffusion of the recently inserted extrasynaptic CP-AMPARs.

### Regulation of GluA2-Containing AMPARs

The observed elevation in extrasynaptic *de novo* GluA1 occurs alongside a reduction in cell-surface *de novo* GluA2 and extant dendritic GluA2/3 while no cell-wide reduction in levels was evident. As GluA2 mRNA is also expressed in dendrites, local downregulation of GluA2 synthesis may be controlled locally via the RNA interacting protein, CPEB3 (Savtchouk et al., 2016) or FMRP (Muddashetty et al., 2007), or via microRNA (miRNA). Indeed, we have previously shown that sAPPα transiently upregulates miR-30 (Ryan et al., 2013), a miRNA known to regulate the expression of GluA2 (Song et al., 2019). Alternatively, association with Protein Interacting with C Kinase-1 (PICK1) may restrict GluA2 trafficking (Terashima et al., 2004; Jaafari et al., 2012) or AP2-GluA2 binding may also promote internalization (Hanley, 2018). Furthermore, the NO-cGMP-PKG and MAPK pathways have been found to be involved in de-clustering of GluA2/3-containing AMPARs (Endo and Launey, 2003), an event which is typically associated with subsequent internalization of receptors (Matsuda et al., 2000). As synaptic protein synthesis, expression of Arc protein, and GluA1 trafficking are dependent on the activity of PKG and MAPK (Claasen et al., 2009; Livingstone et al., 2019; Mockett et al., 2019), sAPPα’s induction of these pathways may also contribute to GluA2/3 receptor internalization. Further, rapid downregulation of GluA2-containing calcium impermeable AMPARs (CI-AMPARs) may facilitate GluA1 cell-surface expression via increasing the available “slots” within the PSD (MacDougall and Fine, 2013) to be filled by CP-AMPARs (Bellone and Lüscher, 2005, 2006; Hong et al., 2013). Indeed, *in vitro* live-cell imaging shows simultaneous downregulation of cell-surface GluA2/3 and upregulation of GluA1-containing AMPARs following LTP induction (Tanaka and Hirano, 2012).

Interestingly, we also observed a delayed increase in the proportion of GluA1/2 AMPARs following sAPPα treatment, at both the somatic and dendritic cell-surface primarily at synaptic sites. These receptors were likely derived from pre-existing pools as they were not tagged in the FUNCAT-PLA assays. Previous work has shown that chronic overexpression of APP *in vitro* upregulates cell-surface GluA2 expression (Lee et al., 2010), indicating that APP, and likely sAPPα, may regulate the persistence of synaptic plasticity through the long-term expression of GluA2-containing AMPARs. As their increase occurs at a timepoint at which both *de novo* GluA1 and extant GluA2/3 are internalized, this would result in a switch to synapses expressing GluA1/2 CI-AMPARs (Shi et al., 2001; Bellone and Lüscher, 2005; Sutton et al., 2006; Mameli et al., 2007), as observed during reconsolidation of memories (MacDougall and Fine, 2013). As we have shown that sAPPα enhances Arc expression and that internalization of *de novo* GluA1 is Arc dependent, Arc expression likely facilitates the addition of synaptic GluA1/2-containing AMPARs (McCormack et al., 2006; Hong et al., 2013). Indeed, previously knockdown of Arc has been shown to increase cell-surface GluA1- but not GluA2-containing AMPARs (Shepherd et al., 2006), while overexpression of Arc protein significantly decreases CP-AMPAR–associated rectification in neurons (DaSilva et al., 2016), a mechanism which may permit addition of CI-AMPARs. Recently, increased Arc expression has been shown to protect neurons against CP-AMPAR–mediated oxyhemoglobin excitotoxicity (Chen et al., 2020), via endocytosis of these AMPARs. While reduced phosphorylation of GluA1_*S*__845_ may also contribute to internalization (Man et al., 2007; Lussier et al., 2015), our observations support a role for Arc-dependent endocytosis in the regulation of *de novo* CP-AMPARs.

Of note, sAPPα has been previously shown to enhance LTP and plasticity-related protein synthesis via a mechanism involving α7nAChRs (Richter et al., 2018; Livingstone et al., 2019) in that antagonism of α7nAChRs during the priming phase, but not during the induction of LTP, impairs the expression of sAPPα-enhanced LTP (Richter et al., 2018). Our current work suggests that this occurs by promoting the synthesis and trafficking of CP-AMPARs. Indeed, activation of postsynaptic α7nAChRs by acute nicotine treatment promotes the rapid trafficking of CP-AMPARs (Lozada et al., 2012; Halff et al., 2014; Tang et al., 2015), while chronic α7nAChR activation enhances the cell-surface expression of GluA1/2 AMPARs (Duan et al., 2015).

## Conclusion

Many pathological changes in Alzheimer’s disease are associated with changes in the expression of AMPA-subtype glutamate receptors, the regulation of which is critical to memory formation. We have shown one mechanism by which sAPPα enhances LTP is by *de novo* synthesis of GluA1-containing CP-AMPARs which are trafficked to extrasynaptic sites. Simultaneously, sAPPα promotes the removal of GluA2/3-containing AMPARs, increasing the number of available synaptic slots, and permitting the trafficking CP-AMPARs from extrasynaptic domains in response to stimulation events which enhance synaptic transmission. The synthesis of Arc protein facilitates removal of synaptic AMPARs which are replaced by GluA1/2-containing CI-AMPARs. Future research should aim to investigate the contribution of Ca^2+^-permeable and GluA2-containing AMPAR to sAPPα-enhanced LTP, and the role of excitatory and inhibitory neurons in this mediating these effects. This work is crucial to understanding the mechanisms harnessed by sAPPα to promote memory formation and further inform the development of alternative treatment strategies for Alzheimer’s disease.

## Data Availability Statement

The original contributions presented in the study are included in the article/Supplementary material, further inquiries can be directed to the corresponding author/s.

## Ethics Statement

The animal study was reviewed and approved by University of Otago Animals Ethics Committee and conducted in accordance with New Zealand Animal Welfare Legislation under the ethics approval ET18/15 and AUP-18-136 for cell culture work and DET19/16 for all acute slice work.

## Author Contributions

RL: was the major contributor to the experimental aspects of the study, contributed to the design of the study, prepared the primary neuronal cultures, carried out immunocytochemistry, FUNCAT-PLA, PLA, and electrophysiology experiments, and analyzed and interpreted the corresponding data and drafted the manuscript. ME: participated in the design and co-ordination of the study and critically assessed the manuscript. AS: participated in the design and co-ordination of the study and critically revised the manuscript. CW: carried out immunocytochemistry and corresponding data analysis. WT: co-ordinated sAPPα and critically revised the manuscript. WA: participated in the design and co-ordination of the study, undertook data analysis and interpretation, and critically revised the manuscript. JW: conceived and participated in the design and co-ordination of the study, undertook data analysis and interpretation, and critically assessed the manuscript. All authors contributed to the article and approved the submitted version.

## Conflict of Interest

The authors declare that the research was conducted in the absence of any commercial or financial relationships that could be construed as a potential conflict of interest.
