# Supplementary material for: Secreted Amyloid Precursor Protein-Alpha Enhances LTP Through the Synthesis and Trafficking of Ca2+-Permeable AMPA Receptors
Source: Front Mol Neurosci. 2021 Apr 1;14:660208. doi: 10.3389/fnmol.2021.660208 (PMC8047154; doi:10.3389/fnmol.2021.660208)
Supplement: Supplementary file 1 [file Table_1.DOCX]

Supplementary Material

# Supplementary Figures and Tables

## Supplementary Figures


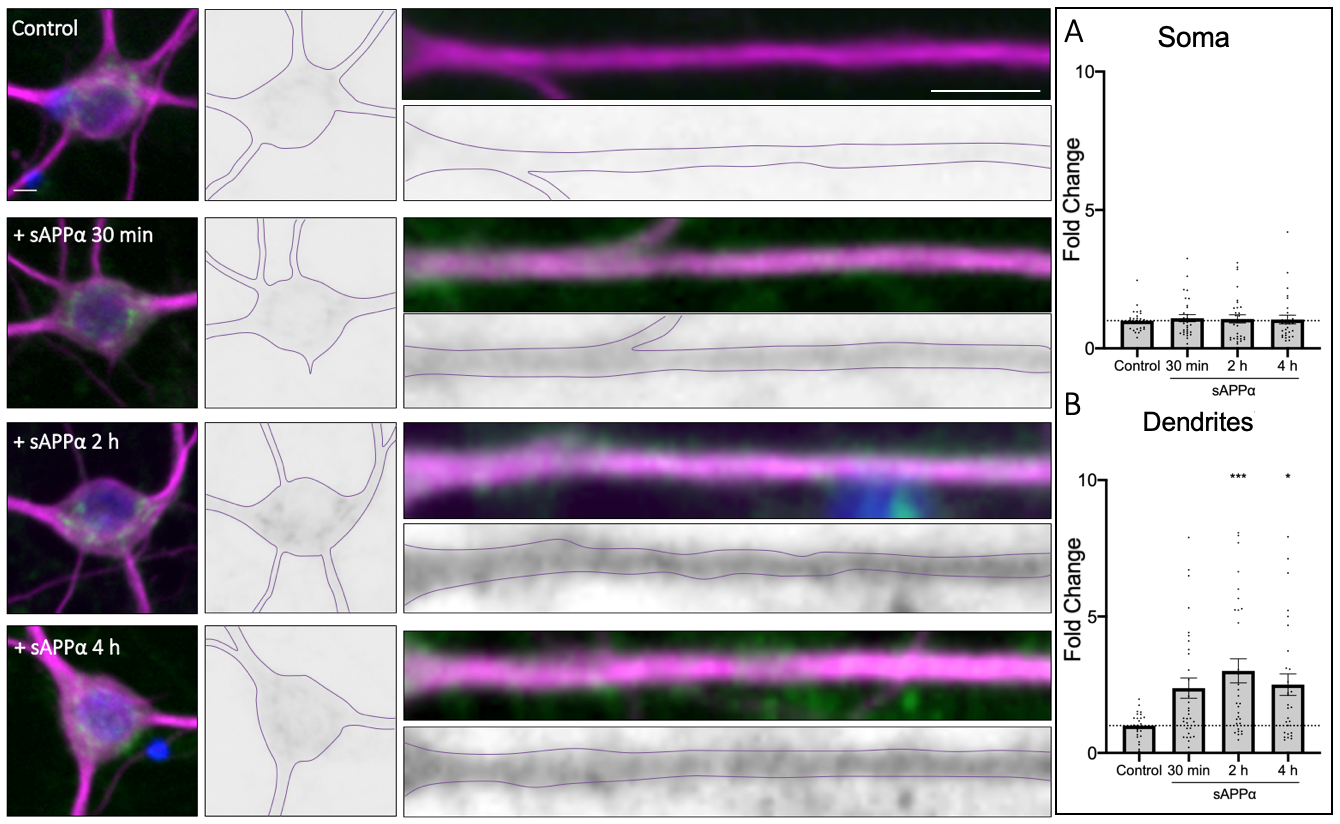


**Supplementary Figure 1.**

**Effect of sAPPα on GluA1 cell surface expression as detected by immunochemistry.**

Representative images and summary bar graphs show cell surface GluA1 in the soma (left panels; A) and dendrites (right panels; B) following treatment with sAPPα for 30 min, 2 h, and 4 h. GluA1 was detected using an *N*-terminal antibody in detergent-free conditions. sAPPα (30 min, 2 h, 4 h; 1 nM) did not affect somatic GluA1 (A) but significantly increased cell surface GluA1 expression in dendrites at 2 and 4 hours (*n* = 28-31). All data are expressed relative to the experimental control from 3 experiments (mean ± SEM). Normality was detected by D’Agostino and Pearson omnibus normality tests and significance was calculated using a Kruskal–Wallis one-way ANOVA with Dunn’s multiple comparisons test on raw data, ^∗∗∗^*p =* 0.0002,  ^∗^*p* = 0.0204. Representative images show neuronal soma, dendrites (MAP2; **■**), GluA1 (**■**), nuclei (DAPI; **■**). Scale bars = 10 μm.

**
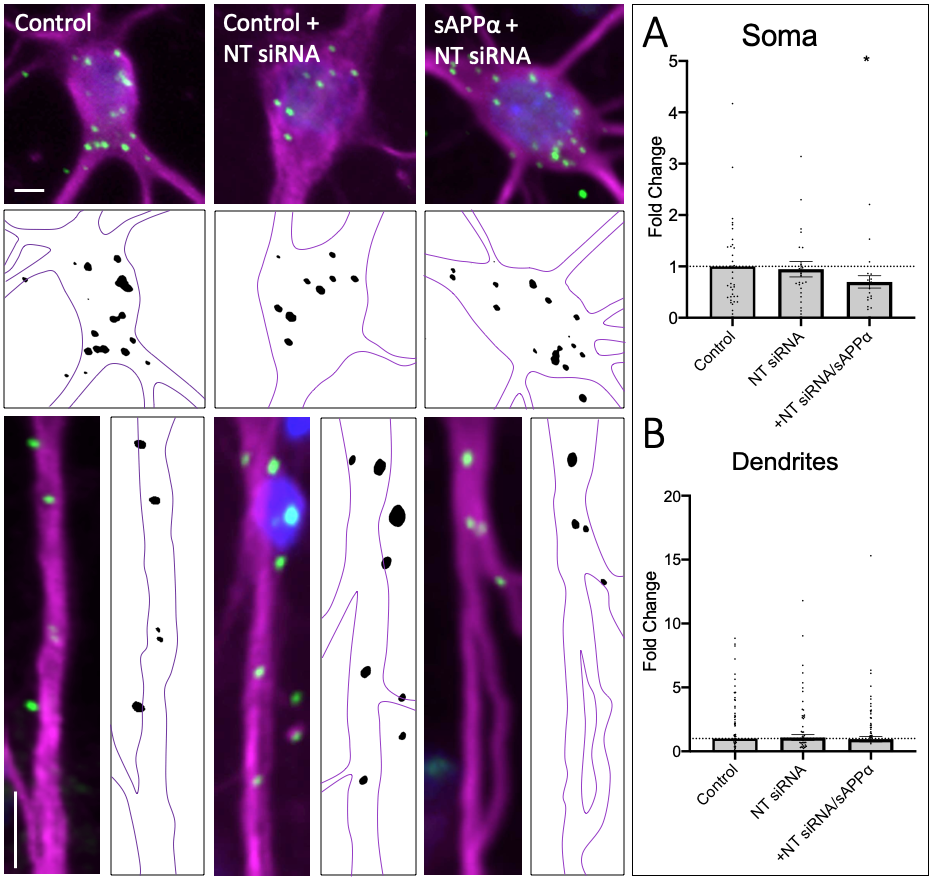
**

**Supplementary Figure 2.**

**Non-targeting siRNA control experiments**

Representative images show cell surface *de novo* GluA1 in the soma (upper panels) and dendrites (lower panels) following treatment with NT siRNA in the presence or absence of sAPPα. Application of non-targeting (NT) control siRNA alone did not significantly affect expression of cell surface *de novo* GluA1, while NT siRNA in the presence of sAPPα resulted in a small but significant decrease in the (A) soma (*n* = 23-24 cells) but not (B) dendrites (*n* = 89-98 dendrites) cell surface GluA1. All data are expressed relative to the experimental control from 3 experiments. Data assessing the effect of siRNA are expressed as mean ratio of sAPPα + NT siRNA/NT siRNA alone ± SEM. Normality was detected by D’Agostino and Pearson omnibus normality tests and significance was calculated using by student’s t-test, ^∗^*p* = 0.0224. Representative images show neuronal soma, dendrites (MAP2; **■**), Arc protein (**■**), nuclei (DAPI; **■**). Scale bars = 10 μm.
